# Discovering and Summarizing Relationships Between Chemicals, Genes, Proteins, and Diseases in PubChem

**DOI:** 10.3389/frma.2021.689059

**Published:** 2021-07-12

**Authors:** Leonid Zaslavsky, Tiejun Cheng, Asta Gindulyte, Siqian He, Sunghwan Kim, Qingliang Li, Paul Thiessen, Bo Yu, Evan E. Bolton

**Affiliations:** National Center for Biotechnology Information, National Library of Medicine, National Institutes of Health, Bethesda, MD, United States

**Keywords:** data mining, knowledge discovery, knowledge summarization, information retrieval, natural language processing, knowledge panels, knowledge graph, PubChem

## Abstract

The literature knowledge panels developed and implemented in PubChem are described. These help to uncover and summarize important relationships between chemicals, genes, proteins, and diseases by analyzing co-occurrences of terms in biomedical literature abstracts. Named entities in PubMed records are matched with chemical names in PubChem, disease names in Medical Subject Headings (MeSH), and gene/protein names in popular gene/protein information resources, and the most closely related entities are identified using statistical analysis and relevance-based sampling. Knowledge panels for the co-occurrence of chemical, disease, and gene/protein entities are included in PubChem Compound, Protein, and Gene pages, summarizing these in a compact form. Statistical methods for removing redundancy and estimating relevance scores are discussed, along with benefits and pitfalls of relying on automated (i.e., not human-curated) methods operating on data from multiple heterogeneous sources.

## Introduction

PubChem (https://pubchem.ncbi.nlm.nih.gov) (Kim et al., 2016a; Kim et al., 2016b; Kim et al., 2019; Kim et al., 2021) is an NIH public repository of chemicals and their biological activities. Along with other NCBI databases (Sayers et al., 2019; Sayers et al., 2021), PubChem provides extensive resources for biomedical discovery. Visited by millions of users every month, PubChem serves a wide range of users, including research scientists, patent agents, chemical hygiene officers, chemical educators, students, and many others. The tremendous growth in the amount of PubChem data, and its increasing heterogeneity and variability in quality, demand a novel exploratory approach to rapidly retrieve relevant, non-redundant, and reliable information and present it in an easy-to-comprehend form, organized around the most useful content for biomedical-focused communities.

PubChem users often want to find and explore important relationships between chemicals, genes, proteins, and diseases, evidenced by peer-reviewed journal articles. This task is not easy, considering the size and scope of the data contained in PubChem. To meet this demand, the literature knowledge panels were developed and implemented in PubChem. For a given entity (i.e., a chemical, gene, or protein), the literature knowledge panels show a few most relevant “neighbors,” which are chemicals, genes, proteins, or diseases mentioned together with the entity. The panels also provide a sample of PubMed records co-mentioning the entity and its neighbors. The information on the relationships between these entities needs to be extracted from public databases, then asserted and summarized. Such a complex collection of interlinked entities is frequently called a knowledge graph (Singhal, 2012; Ehrlinger and Wöß, 2016; Sullivan, 2020; Google, 2021; SciBite, 2021). To make the collection useful, pieces of data relevant to a query need to be found, organized, and presented to the user. In this paper, we describe the methodology that allows us to uncover and summarize important relationships between chemicals, genes, proteins, and diseases by analyzing co-occurrences of terms in the biomedical literature.

The first step in producing data for the literature knowledge panels is to identify relevant named entities in unstructured text. While available data includes trusted curated sets, experimental data provided by various depositors, as well as literature and biomedical publications that are annotated manually by indexers (MEDLINE, 2021); an abundance of data can be extracted from unstructured text using named-entity recognition software (Ratinov, 2009). Current named-entity recognition approaches include dictionary matching, use of rules to recognize specialized terminology, and context analysis using statistical and neural language models (Sayle et al., 2011; Vazquez et al., 2011; Jessop et al., 2012; Rocktäschel et al., 2012; Gurulingappa et al., 2013; Lowe and Sayle, 2015; Pletscher-Frankild et al., 2015; Song et al., 2018; Devlin et al., 2019; Lee et al., 2020; Tian et al., 2020). To produce data for the PubChem literature knowledge panels, entities are annotated in a PubMed record using a third-party named-entity recognition software, LeadMine (Lowe and Sayle, 2015), and matched to chemical synonyms in the PubChem Compound database and to gene, protein, and disease names, as described in *Materials and Methods*.

**FIGURE 1:**
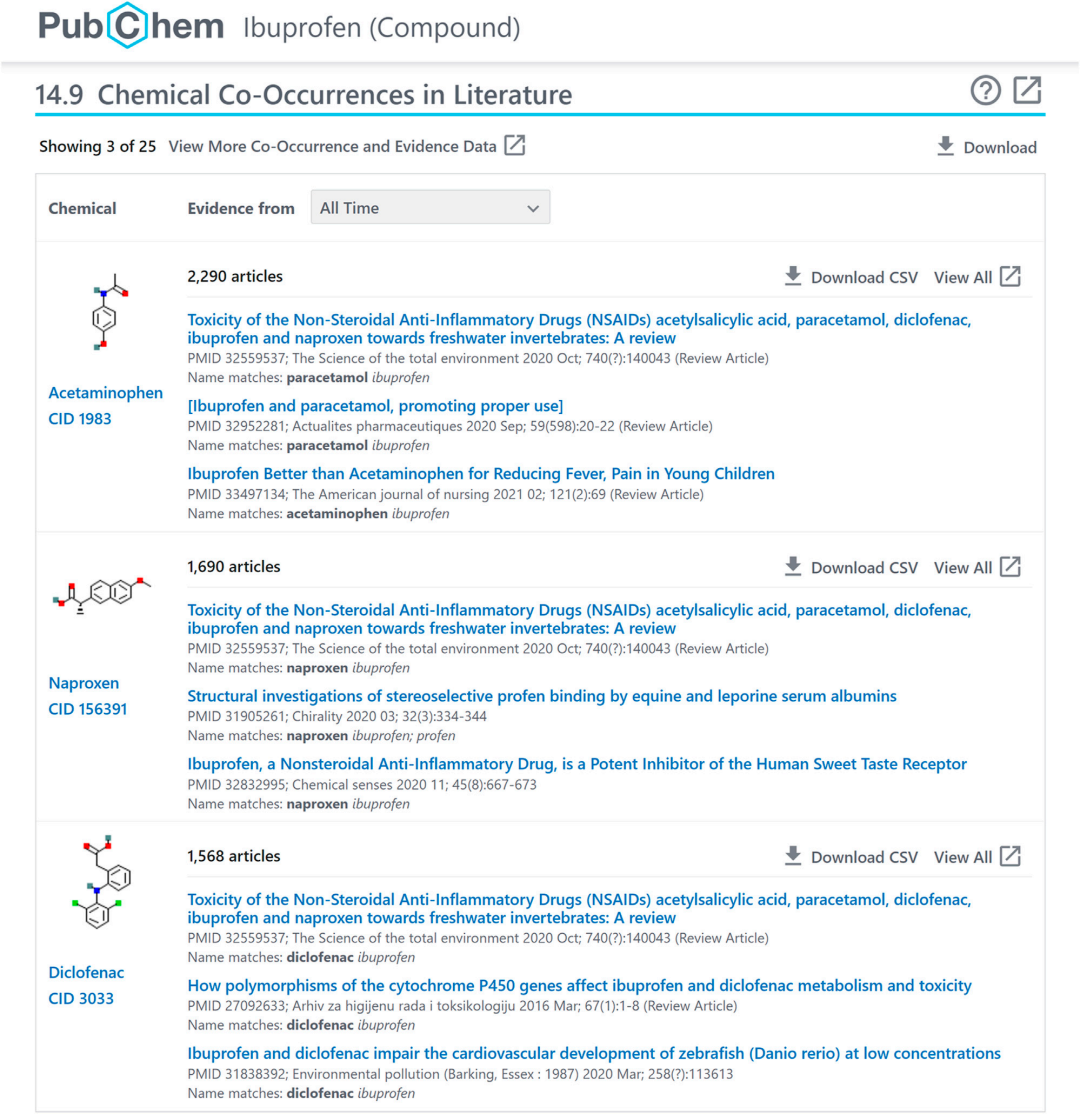
Chemical-chemical co-occurrence panel for ibuprofen (CID 3672), accessible at: https://pubchem.ncbi.nlm.nih.gov/compound/3672#section=Chemical-Co-Occurrences-in-Literature.

**FIGURE 2:**
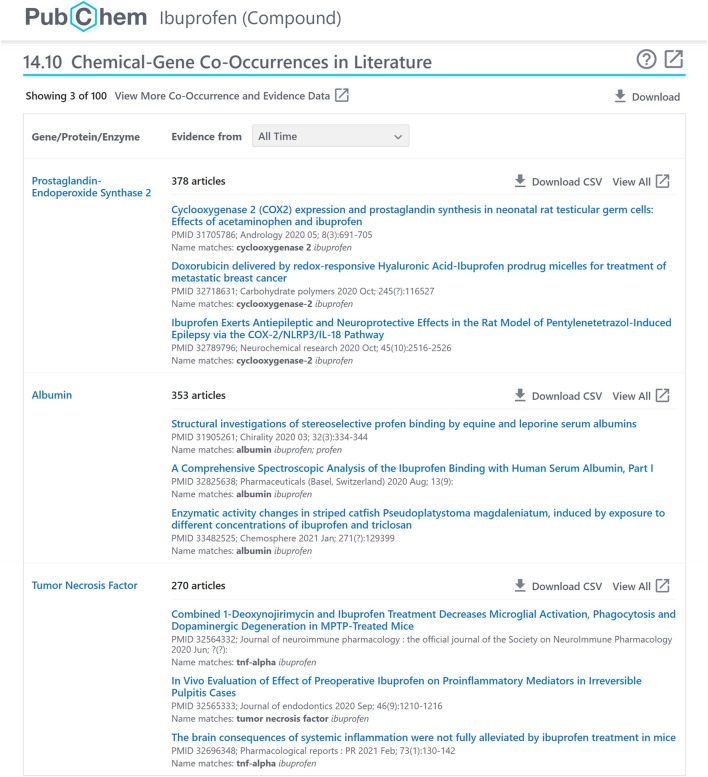
A chemical-gene co-occurrence panel for ibuprofen (CID 3672), accessible at: https://pubchem.ncbi.nlm.nih.gov/compound/3672#section=Chemical-Gene-Co-Occurrences-in-Literature.

**FIGURE 3:**
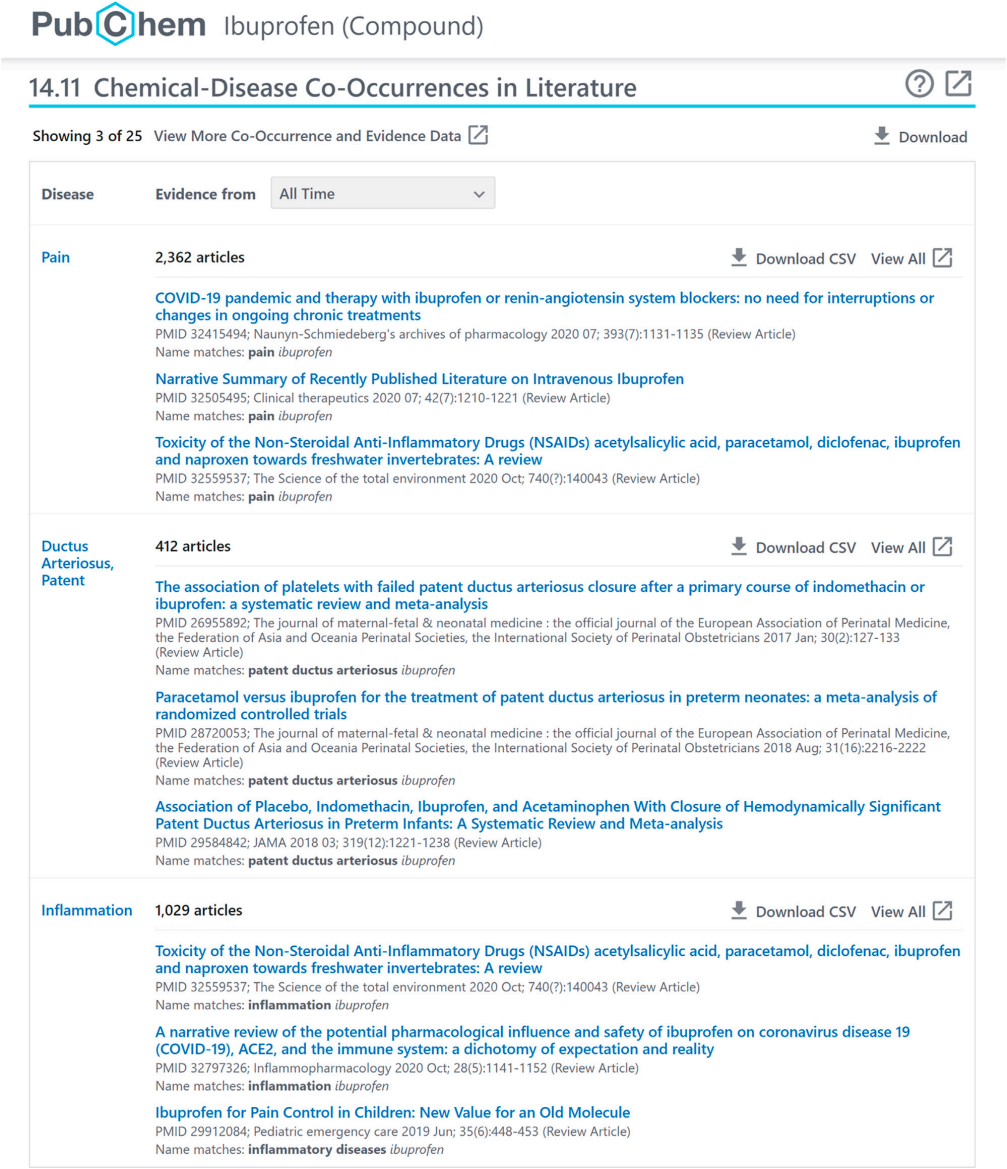
A chemical-disease co-occurrence panel for ibuprofen (CID 3672), accessible at: https://pubchem.ncbi.nlm.nih.gov/compound/3672#section=Chemical-Disease-Co-Occurrences-in-Literature.

The most relevant information is identified through statistical analysis and relevance-based sampling and summarized in a compact form. For each query entity, a few most-relevant neighbors in the knowledge graph are shown, along with several most-relevant PubMed records for each query-neighbor pair, where the query is the entity for which a panel is built (i.e., a compound, gene, or protein) and the neighbor is the co-occurring entity (i.e., a compound, gene, protein, or disease). Additional information accompanying each sample of records as well as download links helps the user to examine the context of the identified relationship and its reliability. The links to examples of literature knowledge panels are listed in Table 1, with screenshots shown in Figures 1–3.

**TABLE 1:** Types of literature co-occurrence panels implemented in PubChem with examples.

Page type	Query ID	Panel type	Link
Compound	CID: 3672	Chemical-chemical	https://pubchem.ncbi.nlm.nih.gov/compound/3672#section=Chemical-Co-Occurrences-in-Literature
Compound	CID: 3672	Chemical-gene	https://pubchem.ncbi.nlm.nih.gov/compound/3672#section=Chemical-Gene-Co-Occurrences-in-Literature
Compound	CID: 3672	Chemical-disease	https://pubchem.ncbi.nlm.nih.gov/compound/3672#section=Chemical-Disease-Co-Occurrences-in-Literature
Target	Gene symbol: ptgs2	Gene-chemical	https://pubchem.ncbi.nlm.nih.gov/gene/ptgs2#section=Gene-Chemical-Co-Occurrences-in-Literature
Target	Gene symbol: ptgs2	Gene-gene	https://pubchem.ncbi.nlm.nih.gov/gene/ptgs2#section=Gene-Gene-Co-Occurrences-in-Literature
Target	Gene symbol: ptgs2	Gene-disease	https://pubchem.ncbi.nlm.nih.gov/gene/ptgs2#section=Gene-Disease-Co-Occurrences-in-Literature

Redundancy and near-redundancy elimination are performed for PubChem compounds using exclusion rules. We discuss three different methods for scoring co-occurrences: the simplest being a score based on the number of records where two entities are co-mentioned, and the other two being more advanced information-based scores that allow to correct for an abundance of the neighbor in the database. To allow some user flexibility, while assuring PubChem efficiency, we enable the user’s selection from a limited number of options, with data precalculated for each option.

The details of our methodology, data sources, and implementation are described in *Materials and Methods*. The application of the methodology to the real data is discussed in *Results*. The limitations of the current approach plus opportunities to enhance and extend using information from trusted human-curated sources as well as a variety of heterogeneous data sources are discussed in *Discussion*.

## Materials and Methods

NCBI PubMed records (PubMed, 2021; Sayers et al., 2021) are downloaded, and annotated using LeadMine, an entity recognition software program that uses dictionary matching and rules for specialized chemical terminology (“grammar”) to recognize relevant text entities (Lowe and Sayle, 2015), version 3.15. Annotation is performed in multiple categories (such as chemicals, genes, proteins, and diseases) using LeadMine-provided dictionaries as well as our in-house dictionaries. While nested annotations (such as a chemical name, like “salicylic acid,” found in a protein name, like “salicylic acid-binding protein 2”) are allowed, only the non-nested annotations are used to build the knowledge panels. Annotated entities nested inside other annotated entities are kept for internal use, such as quality control and disambiguation.

The entities identified in PubMed records are normalized and matched to PubChem Compound database synonyms. Similarly, gene names and protein names are matched to the respective PubChem Gene and PubChem Protein pages, respectively, through the process described below. Disease entities are matched to Medical Subject Headings (MeSH) headers and supplementary concepts (MeSH, 2021) using LeadMine. For each query entity (a compound, gene, or protein), several non-redundant neighbors (compounds, diseases, genes, or proteins) are selected based on the co-occurrence scores between the query and neighbors. The scores depend upon the counts of PubMed records co-mentioning the query-neighbor pair. PubMed records co-mentioning them are sampled based on the relevance score, which reflects the position and frequency of the co-mentioned entities in the PubMed records as well as the characteristics of the PubMed records (e.g., the article type and publication date recency). The matching algorithms and scoring schemes are discussed in more detail below.

### Text Entity Matching

Disease text entities are matched to MeSH headers and supplementary concepts using dictionaries and resolvers provided by LeadMine (Lowe et al., 2016), with some corrections made to accommodate recent changes in MeSH. Other annotated entities are matched to the names of chemicals, genes, and proteins, using the matching algorithm described in the paragraphs below.

While the entities annotated using LeadMine’s case-insensitive dictionaries are matched in a case-insensitive manner, capitalization is considered when matching entities annotated by LeadMine using case-sensitive dictionaries. There is a normalization step performed, where all brackets become round brackets. In addition, before matching, text entities and database entries are transformed to ASCII (from UTF-8 or Unicode character sets), when possible, using the functionality from the Open Parser for Systematic IUPAC Nomenclature (OPSIN) project (Lowe et al., 2011).

Entities are considered matched if they have the same alphanumeric string (also using case-sensitive matching for alphanumeric strings produced from the entities annotated with case-sensitive dictionaries, and case-insensitive otherwise) and, if there is a high alignment score, allowing some flexibility in non-alphanumeric symbols. A pair of the text entities with identical alphanumeric strings are aligned using the Needleman–Wunsch algorithm (Needleman and Wunsch, 1970) with weight: 1 for exact matches, and −1 for mismatches and gaps. For two aligned entities to be accepted, the number of matched characters, normalized by the maximum of the lengths of the entities, should be greater than or equal to an acceptance threshold of 0.9 for compounds and 0.7 for genes and proteins. These thresholds were established empirically after experimentation with various cases.

Currently, it is not yet possible to reliably connect gene or protein entities in an unstructured text (e.g., PubMed records) to organism information. When annotating gene and protein entities, LeadMine frequently resolves their names to ones in an obscure organism (e.g., old names of human genes and proteins are resolved to current names in other species). We decided to prioritize human genes and proteins. The following strategy has been implemented to resolve gene and protein text entities to the most reasonable gene, protein, or enzyme symbol (corresponding to human, when possible):

- Try to find a match among Human Genome Organization (HUGO) Gene Nomenclature Committee (HGNC) names (Braschi et al., 2019; HUGO, 2021);
- Try to find a match among names in The IUPHAR/BPS Guide to Pharmacology (Armstrong et al., 2020; IUPHAR/BPS, 2021);
- Try to find matches among names in UniProt (Bateman et al., 2017);
- Otherwise, try to match to an enzyme name and resolve to an EC number (Bairoch, 2000; Expassy, 2021).

In general, it is very difficult and often impossible to distinguish the name of a gene from the name of the protein encoded by that gene. Therefore, gene and protein names are not strictly distinguished from each other but considered as one category. Therefore, the annotations considered in this study can be grouped into three categories: chemicals, genes/proteins, and diseases.

### Relevance Score for a Pair of Entities in a PubMed Record

The relevance score for two entities co-mentioned in a PubMed record is used to sample PubMed records that may provide a context as to their relationship. The relevance scoring scheme has been carefully crafted to reflect the following factors:

- The occurrence of the query-neighbor pair in the title significantly increases the relevance of the publication;
- Annotated entities that appear close in the text have more chance to be related (Manning and Schütze, 1999);
- With all other factors being the same, a recent publication is probably more important than an old one;
- The PubMed record corresponding to a “review” article slightly increases the relevance of the publication.

The relevance score $r_{\mathrm{ij}}^{p}$ for matched entities i and j in PubMed record p  is calculated by the following empirical formula

$$r_{\mathrm{ij}}^{p}=\;w_{T}\delta T_{i}^{p}\delta T_{j}^{p}+w_{S}\delta S_{\mathrm{ij}}^{p}+\;w_{M}\left(1+\delta T_{i}^{p}+\;\delta N_{i}^{p}\right)\left(1+\delta T_{j}^{p}+\;\delta N_{j}^{p}\right)+\;w_{R}\delta R^{p}+w_{A}\delta A^{p},$$ (1)

where,

$\delta T_{i}^{p}$ is 1 if the matched entity i is in the title of the record p, and is 0 otherwise ($\delta T_{j}^{p}$ is defined similarly);

$\delta N_{i}^{p}$ is 1 if the matched entity i is present in the record p more than once, and is 0 otherwise ($\delta N_{j}^{p}$ is defined similarly);

$\delta S_{\mathrm{ij}}^{p}$ is 2 if both entities i and j are present in two or more sentences in the abstract, 1 if only in one sentence, and 0 otherwise;

$\delta R^{p}$ is 1 if the record p is marked as corresponding to a review article, and is 0 otherwise;

$\delta A^{p}$ is equal to:

$w_{A\_1}$ if the publication age is within one year;
$w_{A\_2}$ if the publication age is between one and two years;
$w_{A\_5}$ if the publication age is between two and five years;
$w_{A\_10}$ if the publication age is between five and 10 years;
$w_{A\_15}$ if the publication age is between ten and 15 years;
$w_{A\_20}$ if the publication age is between fifteen and 20 years;
0 if the publication age is more than 20 years.

Weights used in Eq. 1 were selected after careful consideration. The values currently used in PubChem production are:$\;w_{T}=50$ $w_{S}=50$; $w_{M}=10$; $w_{R}=10$; $w_{A\_1}=25$; $w_{A\_2}=20$; $w_{A\_5}=15$; $w_{A\_10}=10$; $w_{A\_15}=5$; $w_{A\_20}=2$.

Eq. 1 and the values of the weights have been established by looking into a variety of representative PubMed records and by establishing the relative importance of the contributing factors. It is difficult to objectively check the accuracy and reliability of the formula and weights because of the subjectivity of entity relationship interpretation and relative scarcity of curated data. Still, although the formula is heuristic and the weights values are subjective, they could be further optimized to handle specific use cases.

### Selecting the Time Period for the Publication Dates

While a reconciliation of different relevance factors is an intricate problem, balancing the publication date with other relevance factors can be especially difficult, and strongly depends on the user’s needs. While operating with the pre-calculated data in the default setting, the user can select a preferred publication time period from a limited number of options (currently three options: since last year, within the past 5 years, or within the past 10 years). Based on the user’s selection, the page view is formed within the web browser from the pre-calculated data. This approach allows some flexibility while assuring system efficiency.

### Scoring the Co-occurrences

The co-occurrence score between two entities is used to select the most co-mentioned entities for a given entity. Three approaches have been tested to develop an appropriate formula for the co-occurrence score.

Consider the query entity i and the neighbor entity j, which belong to the categories *I* and *II*, respectively, and let $\Lambda^{\left(I\right)}$and $\Lambda^{\left(\mathrm{II}\right)}$ be sets of PubChem records that have mentions from the categories *I* and *II*, respectively, and $\Lambda^{\left(I,\mathrm{II}\right)}=\Lambda^{\left(I\right)}\cap \Lambda^{\left(\mathrm{II}\right)}$ **.** Let $\Lambda_{i}^{\left(I\right)}\subseteq \Lambda^{\left(I\right)}$ and $\Lambda_{j}^{\left(\mathrm{II}\right)}\subseteq \Lambda^{\left(\mathrm{II}\right)}$ be sets of PubChem records mentioning entities i and j, $\;\Omega_{i}^{\left(I\right)}=\Lambda_{i}^{\left(I\right)}\cap \Lambda^{\left(I,\mathrm{II}\right)}$, and $\Omega_{j}^{\left(\mathrm{II}\right)}=\Lambda_{j}^{\left(\mathrm{II}\right)}\cap \Lambda^{\left(I,\mathrm{II}\right)}$. Denote $\Omega_{\mathrm{ij}}=\;\Omega_{i}^{\left(I\right)}\cap$ $\Omega_{j}^{\left(\mathrm{II}\right)}$ be a set of PubChem records where entities i and j are co-mentioned (it is easy to see that $\Omega_{\mathrm{ij}}=\;\Lambda_{i}^{\left(I\right)}\cap$ $\Lambda_{j}^{\left(\mathrm{II}\right)}$ as well).

We considered the following choices for the co-occurrence score $S_{\mathrm{ij}}$ **.** First, we used

$$S_{\mathrm{ij}}=\;N_{\mathrm{ij}},$$ (2)

where $N_{\mathrm{ij}}=|\Omega_{\mathrm{ij}}|$ **.** This simple scoring scheme is suitable when the neighbor j is relatively rarely present in the dataset $\Lambda^{\left(I,\mathrm{II}\right)}$. However, when the neighbor j frequently occurs in the PubMed articles (e.g., the chemical name “water” or the disease term “cancer”), this scheme tends to bring it to the top of the neighbor list of entity i, even if the relationship between i and j is not very specific. This can be avoided by switching to the information gain-based co-occurrence score calculated by the formula

$$S_{\mathrm{ij}}=\;N_{\mathrm{ij}}\left(1-\;\frac{\log\;N_{j}}{\log\;N_{\mathrm{DS}}}\right),$$ (3)

where $N_{\mathrm{DS}}=|{\text{Λ}}^{\left(I,II\right)}|\;$is the size of the dataset, and $N_{j}=|\Omega_{j}^{\left(\mathrm{II}\right)}|$ is the number of records within $\Lambda^{\left(I,\mathrm{II}\right)}\;$where entity j is mentioned. The score (3) is derived from the Kullback–Leibler divergence, also known as relative entropy (Kullback and Leibler, 1951; Manning et al., 2008). It can be considered as a variant of term frequency–inverse document frequency (TF-IDF) score (Aizawa, 2003; Robertson, 2004; Manning et al., 2008; Rajaraman and Ullman, 2011). At the time of writing, Eq. 3 is what is used by PubChem co-occurrence displays.

To define an even more advanced scoring formula, let us denote the relevance score of the entities i and j in the record $p\in \Omega_{\mathrm{ij}}$ as $r_{\mathrm{ij}}^{p}$ **,**

$${\bar{r}}_{j}^{p}=\max_{i}\left\{r_{\mathrm{ij}\;}^{p}\text{|}\;\Omega_{\mathrm{ij}}\neq \emptyset \;\wedge p\in \Omega_{\mathrm{ij}}\right\},$$

and

$${\bar{N}}_{j}\left(\alpha \right)=\left|\left\{p\in \Omega_{j}^{\left(\mathrm{II}\right)}\;\text{|}\;{\bar{r}}_{j}^{p}\geq \alpha \right\}\right|.$$

Then the co-occurrence score is defined by the formula

$$S_{\mathrm{ij}}=\sum_{p\in \Omega_{\mathrm{ij}}}\left(1-\;\frac{\log\;{\bar{N}}_{j}\left(r_{\mathrm{ij}}^{p}\right)}{\log\;N_{\mathrm{DS}}}\right)\;.$$ (4)

While co-occurrence scores defined by Eqs 2, 3 depend only on article counts, the score defined by Eq. 4 also depends on the distribution over relevance score. Further explanation on these scoring schemes is provided in *Results*.

### Redundancy Elimination

Some compounds in PubChem are very similar (e.g., different salt forms of the same parent compound), and if such compounds happen to be neighbors to the query compound in the knowledge graph, the panel will be clogged with redundant information, decreasing its utility. This redundancy was removed by selecting a representative neighbor from each “group” of neighbors with either the same parent-connectivity group (Fu et al., 2015) or (more selectively) the same chemical name. The representative neighbors are selected using these rules:

- All compounds that belong to the same parent-connectivity group or have the same name as the query compound are taken out of consideration;
- The same rule is iteratively applied when PubChem compounds are added to the knowledge panel as neighbor compounds. At each iteration, the compound with the highest value of co-occurrence score (based on Eq. 3) is selected from the list of candidates (in the case of the same value of co-occurrence score, the selection is arbitrary). The selected compound is added to the knowledge panel as a representative neighbor compound, while all compounds that belong to the same parent-connectivity group as that compound or share a name with it are removed from the list (only names from the PubChem list of synonyms that matched PubMed records are taken into consideration).

The application of these rules results in a “non-redundant” neighbor list for a query entity. Note that these rules ensure that neighbors are not too similar to each other as well as to the query.

### Implementation

The precalculated co-occurrence data are loaded into a set of databases and served to the knowledge panels presented in the respective literature sections of PubChem Compound, Gene, and Protein pages. When the summary page for a given PubChem record is created, these databases are queried to see if there is co-occurrence information for the specific record in question. If so, an appropriate heading is added to the summary (in the Literature section of the table of contents). When the user scrolls to that part of the summary page, the databases are queried to gather the information displayed in the panels.

As with many other NLP-based tools, we do not distinguish protein names from the names of the encoding genes since they are frequently used interchangeably. Examples of the six types of literature co-occurrence panels are shown in Table 1.

**FIGURE 4:**
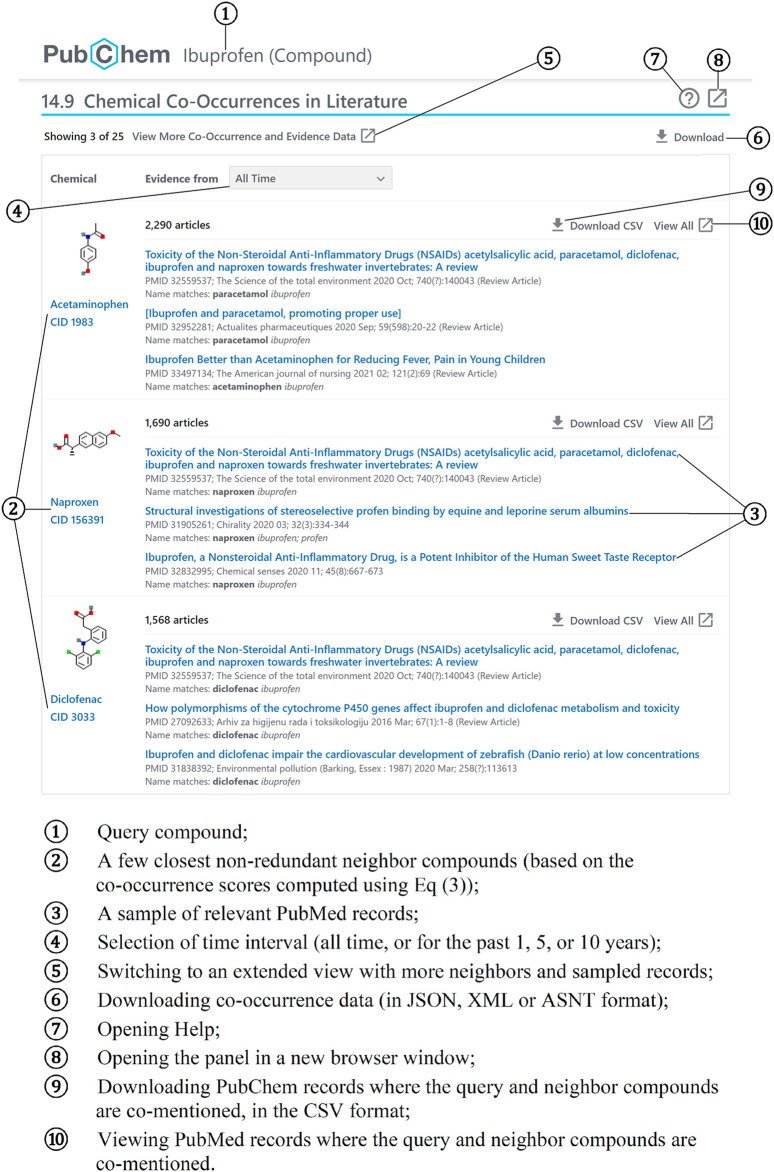
Information and control options in the chemical co-occurrence panel for ibuprofen.

Figure 4 shows the chemical co-occurrence panel for ibuprofen (https://pubchem.ncbi.nlm.nih.gov/compound/3672#section=Chemical-Co-Occurrences-in-Literature) with the annotation of the information included in the panel and control options, which help the user to examine the context and reliability of the relationships:

① Query compound;

② A few closest non-redundant neighbor compounds (based on the co-occurrence scores computed using Eq. 3) with links to corresponding records (e.g., https://pubchem.ncbi.nlm.nih.gov/compound/1983);

③ A sample of relevant PubMed records with links (e.g., https://www.ncbi.nlm.nih.gov/pubmed/32559537);

④ Selection of time interval (all time, or for the past 1, 5, or 10 years);

⑤ Switching to an extended view with more neighbors and sampled records (e.g., https://pubchem.ncbi.nlm.nih.gov/compound/3672#section=Chemical-Co-Occurrences-in-Literature&fullscreen=true);

⑥ Downloading co-occurrence data in JSON, XML or ASNT format (e.g., https://pubchem.ncbi.nlm.nih.gov/link_db/link_db_server.cgi?format=JSON&type=ChemicalNeighbor&operation=GetAllLinks&id_1=3672&response_type=display);

⑦ Opening Help;

⑧ Opening the panel in a new browser window (e.g., https://pubchem.ncbi.nlm.nih.gov/compound/3672#section=Chemical-Co-Occurrences-in-Literature&fullscreen=true);

⑨ Downloading PubChem records where the query and neighbor compounds are co-mentioned, in the CSV format (e.g., https://pubchem.ncbi.nlm.nih.gov/link_db/link_db_server.cgi?response_type=save&type=ChemicalNeighborAll&operation=GetCSV&id_1=3672&id_2=1983);

⑩ Viewing PubMed records where the query and neighbor compounds are co-mentioned (e.g., https://pubchem.ncbi.nlm.nih.gov/link_db/link_db_server.cgi?type=ChemicalNeighborAll&operation=RedirectToEntrez&id_1=3672&id_2=1983).

The data underlying the literature knowledge panels are routinely updated on a weekly basis. The data presented in the next section was generated in late February 2021.

## Results

General statistics for the annotation and matching of PubMed records are shown in Table 2. Among 32.2M PubMed records (as of late February 2021), there are 14.0M that have a chemical annotation, with 11.42M records having a chemical annotation matched to a PubChem compound and 294.6K PubChem compounds matched to PubMed records. Note that nearly all disease terms in the disease dictionaries, including all levels of the MeSH trees, are resolved to MeSH headers and supplementary concept records (Lowe et al., 2016).

**TABLE 2:** General statistics (as of February 27, 2021).

Category	#records	#records with a matched entity in the category	Portion of records that have a matched entity	#unique identifiers
Active records	32.17M	n/a	n/a	n/a
Active records with an annotation	27.34M	23.24M	85.0%	359.75K
Active records having chemical annotations	13.91M	11.42M	81.8%	294.60K
#active records having disease annotations	17.43M	17.41M	99.9%	8.88K
#active records having gene, protein or enzyme annotations	8.73M	6.54M	74.9%	56.28K

**FIGURE 5:**
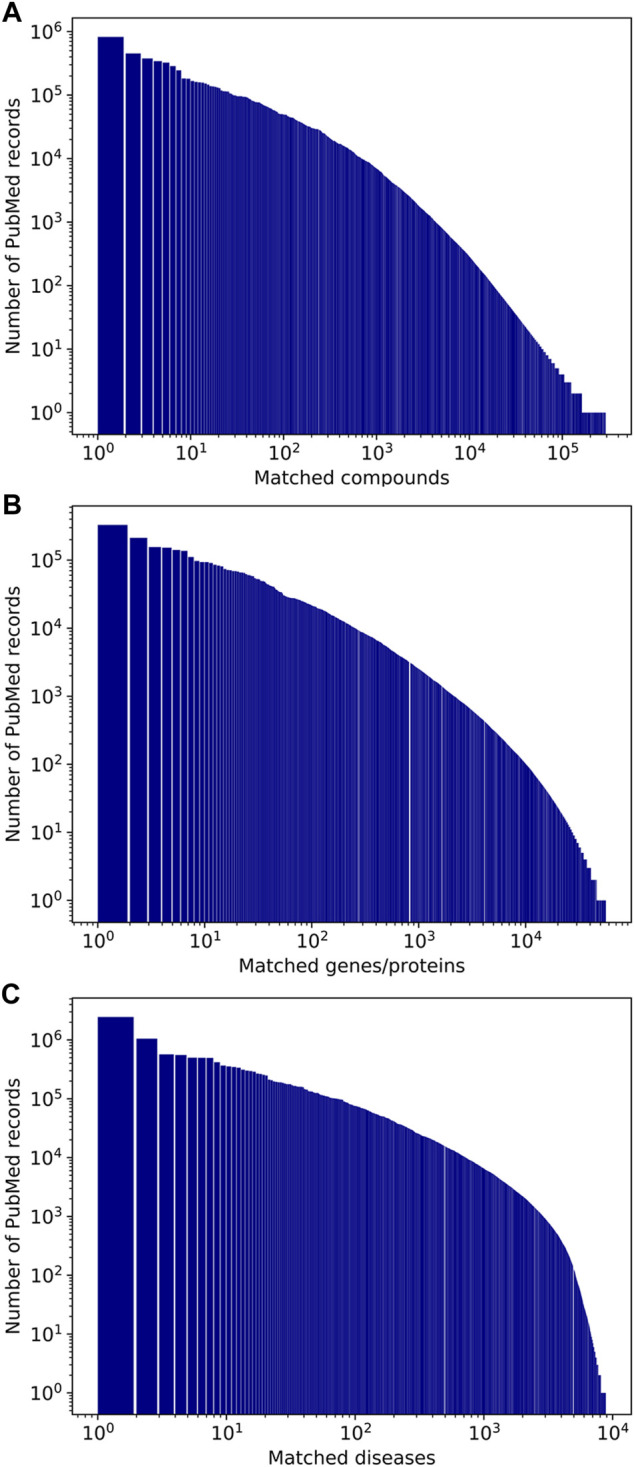
Number of occurrences of compounds (A), genes/proteins (B), and diseases (C) in PubMed records.

The distributions of the occurrences of compounds, genes/proteins, and diseases in PubMed record annotations are illustrated in Figure 5. The five most frequently mentioned entities in PubMed records for the three categories are listed in Tables 3–5. Note that most of the annotations are for a small number of frequently mentioned entities. For example, 79.9% of unique CID-PMID pairs contain only 1% of the 294.6K CIDs. The five most frequently occurring chemicals are water (CID 962), D-glucose (CID 5793), oxygen (CID 977), ethanol (CID 702), and calcium (CID 5460341). Especially, water is annotated in 823.7K PubMed records, which corresponds to 5.9% of all the PubMed records annotated with chemicals and 2.6% of all PubMed records. The most frequently mentioned entities in the gene/protein and disease categories were insulin and neoplasms, respectively, which appeared in 329.4K and 2.46M PubMed records, respectively.

**TABLE 3:** Top five most mentioned chemicals in PubMed records and the number of their chemical, gene/protein, and disease neighbors.

CID	Chemical name	# PubMed records	# Chemical neighbors (non-redundant)	# Gene/protein neighbors	# Disease neighbors
962	Water	823,657	47,183	15,934	4,538
5793	D-glucose	452,960	23,945	16,959	4.303
977	Oxygen	376,484	27,511	12,300	4,037
702	Ethanol	342,100	29,050	11,464	4,172
5460341	Calcium	324,490	17,402	13,408	4,258

**FIGURE 6:**
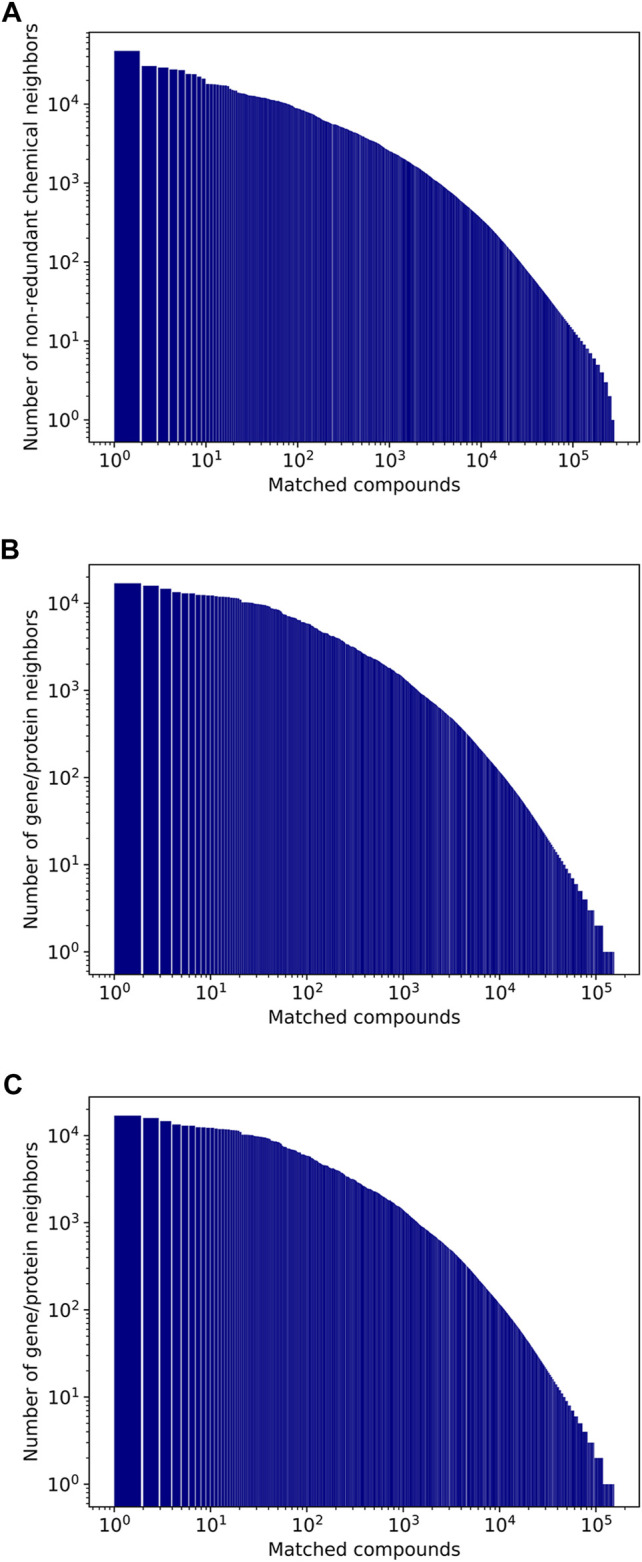
Number of non-redundant neighbors of compounds (A), genes/proteins (B), and diseases (C).

Figure 6 shows the distributions of the number of neighbors for chemicals, genes/proteins, and diseases (the chemical neighbor counts are for non-redundant chemical neighbors, generated using the method described in *Redundancy elimination*). Because frequently mentioned entities are likely to occur with other entities, they often have tens of thousands of neighbors. For instance, the most mentioned chemical, water, has 47.2K non-redundant chemical neighbors, 15.9K gene/protein neighbors, and 4.5K disease neighbors (Table 3). For the gene/protein category, insulin is most frequently mentioned, and has 13.4K non-redundant chemical neighbors, 12.3K gene/protein neighbors, and 3.9K disease neighbors (Table 4). The most mentioned disease term, “neoplasms,” appears with 47.1K non-redundant chemical neighbors, 28.3K gene/protein neighbors, and 6.0K disease neighbors (Table 5).

**TABLE 4:** Top five most mentioned genes/proteins in PubMed records and the number of their chemical, gene/protein, and disease neighbors.

Symbol	Name	# PubMed records	# Chemical neighbors (non-redundant)	# gene/protein neighbors	# Disease neighbors
Ins	Insulin	329,358	13.4K	12.3K	3.9K
Tnf	Tumor necrosis factor	212,766	14.1K	11.9K	3.7K
cd4	CD4 (cluster of differentiation 4)	155,735	6.3K	8.4K	3.4K
Alb	Albumin	152,666	15.2K	7.6K	3.5K
il6	Interleukin 6	141,371	10.6K	10.1K	3.5K

**TABLE 5:** Top five most mentioned diseases in PubMed records and the number of their chemical, gene/protein, and disease neighbors.

MeSH ID	Name	# PubMed records	# Chemical neighbors (non-redundant)	# gene/protein neighbors	# Disease neighbors
D009369	Neoplasms	2,455,851	47.1K	28.3K	6.0K
D007239	Infections	1,050,141	20.2K	20.3K	5.6K
D007249	Inflammation	567,355	21.0K	16.2K	5.3K
D064420	Drug-related side effects and adverse reactions	554,313	48.4K	16.3K	4.7K
D003920	Diabetes mellitus	499,870	13.7K	11.7K	4.7K

Importantly, the frequently mentioned entities tend to be ranked higher in a neighbor list when the co-occurrence scores are computed using Eq. 2. This bias is addressed by using the information gain-based co-occurrence scoring schemes, Eqs 3, 4. To illustrate the importance of correction, consider the chemical co-occurrence panel for acetone (CID: 180):

https://pubchem.ncbi.nlm.nih.gov/compound/180#section=Chemical-Co-Occurrences-in-Literature.

The three closest non-redundant chemical neighbors for acetone are methanol (CID 887), ethanol (CID 702), and water (CID 962). Note that, water is listed as the third closest, although it was more frequently co-mentioned with acetone than the other two neighbors (4.59K records for water, 3.30K for methanol, and 4.3K for ethanol). This is because of the correction term in Eq. 3.

**FIGURE 7:**
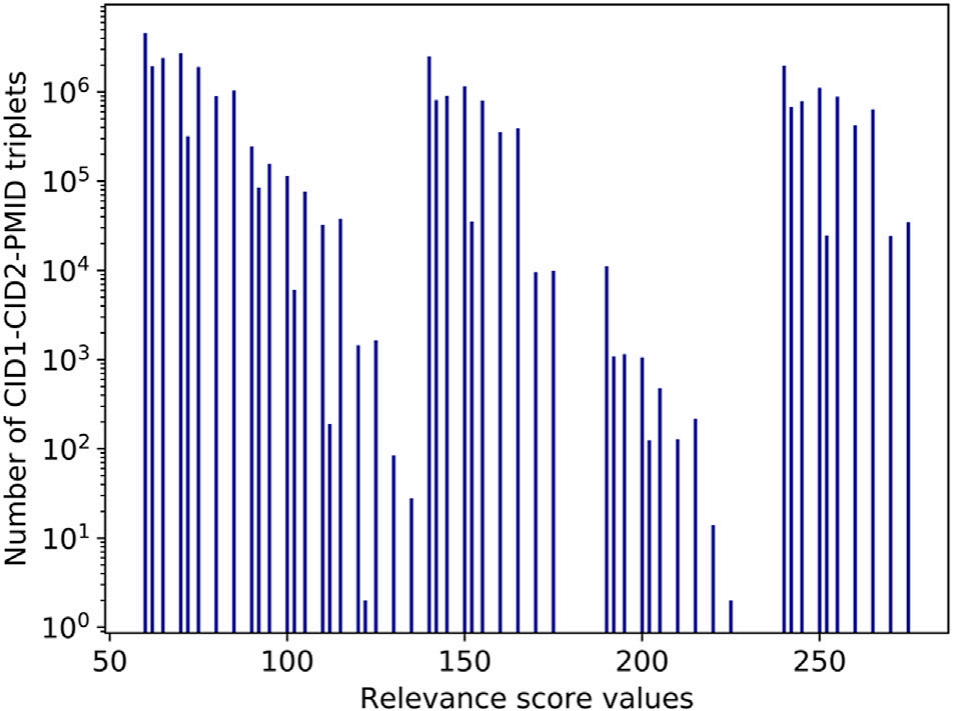
Histogram of the relevance score values.

**FIGURE 8:**
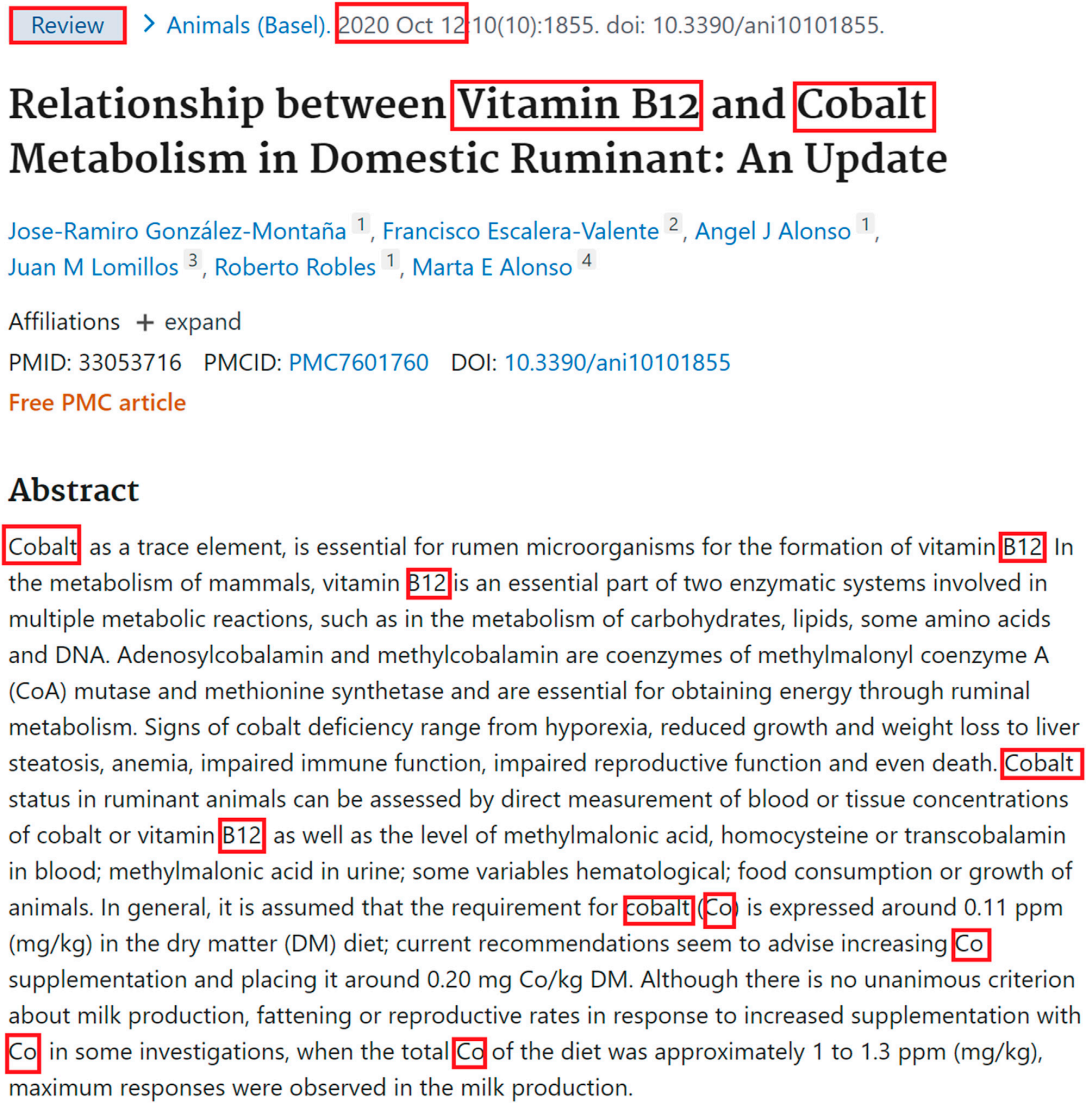
Annotations of vitamin B2 and cobalt in PMID 33053716 “*Relationship between Vitamin B12 and Cobalt Metabolism in Domestic Ruminant: An Update*”.

Figure 7 illustrates the distribution of relevance score values for the CID1-CID2-PMID triplets. Note that the group of columns on the right accounts for the most significant co-occurrences: score values above 240 are produced typically when two compounds are annotated in the title and are mentioned together in two or more sentences in the abstract. For example, vitamin B2 and cobalt have a very high relevance score of 275 in PMID 33053716, with title: “Relationship between Vitamin B12 and Cobalt Metabolism in Domestic Ruminant: An Update” (Gonzalez-Montana et al., 2020), because all factors listed in *Relevance score for a pair of entities in a PubMed record* contribute to the relevance score, as illustrated by Figure 8. The two chemicals appear together in the title as well as in multiple sentences in the abstract. Besides, the paper is a recent review article published a year ago. Important, but fewer significant co-occurrence patterns produce relevance scores in the range 140–240, which correspond to the middle group in Figure 7. Score values in the range 140–240 are produced typically when two compounds are annotated in the title and are mentioned together in one sentence in the abstract.

To understand and compare Eqs 3, 4, let us rewrite them in an alternative form. Eq. 3 can be written as

$$S_{\mathrm{ij}}=\;\vartheta_{j}N_{\mathrm{ij}},$$ (5a)

where the correction factor $\vartheta_{j}$ is defined as

$$\vartheta_{j}=1-\;\varepsilon_{j},$$ (5b)

and

$$\varepsilon_{j}=\;\frac{\log\;N_{j}}{\log\;N_{\mathrm{DS}}}.$$ (5c)

**FIGURE 9:**
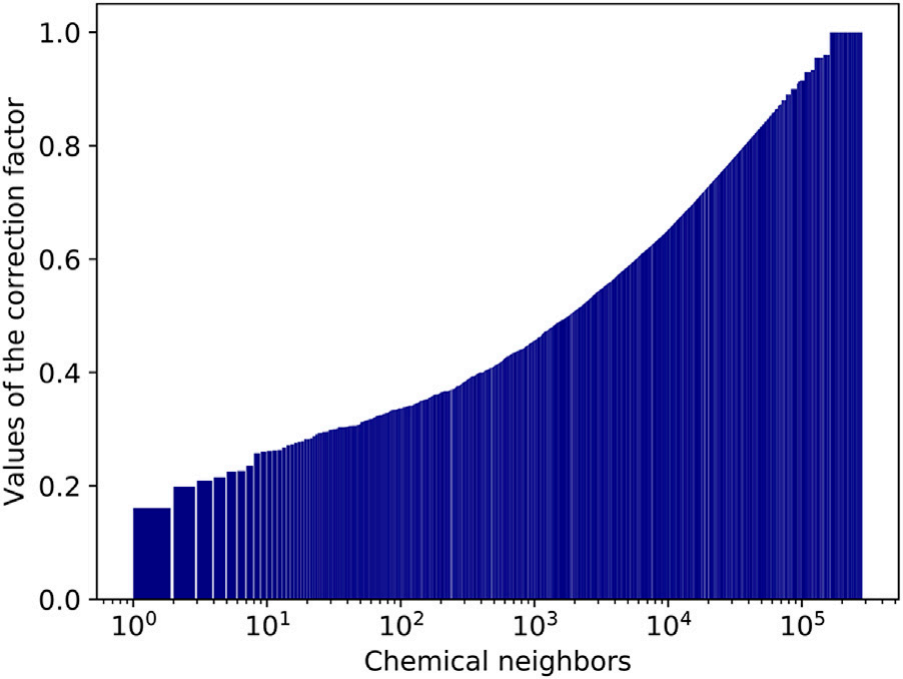
Values of the correction factor $\vartheta_{j}$ for chemical neighbors.

The value of the correction factor $\vartheta_{j}\;$ in Eq. 5a is significantly below 1 when the neighbor j is well-presented in the dataset (i.e., frequently mentioned in PubMed articles) and $\log\;N_{j}$ is comparable to $\log\;N_{\mathrm{DS}}$. For $N_{\mathrm{DS}}=11.42\text{K}$ (the number of PubMed records with matched chemical annotations; Table 3), $\vartheta_{j}=\frac{1}{2}$ when $N_{j}=3.38K$, and $\vartheta_{j}=\frac{1}{3}$ when $N_{j}=50.7\text{K}$. There are about 1.78K compounds whose $\vartheta_{j}\;$values are 1/2 or below. Among them, 82 compounds have $\vartheta_{j}\;$values 1/3 or below. Water has the smallest $\vartheta_{j}\;$ value, equal to 0.161. The values of the correction factor$\;\vartheta_{j}$ in Eq. 5a for chemical neighbors are shown in Figure 9.

Similarly to Eq. 3, Eq. 4, can be written in the form

$$S_{\mathrm{ij}}=\;\vartheta_{ij}^{\prime }N_{\mathrm{ij}},$$ (6a)

where the correction factor $\vartheta_{\mathrm{ij}}^{\prime }$ is defined as

$$\vartheta_{ij}^{\prime }=\;\frac{1}{N_{\mathrm{ij}}}\sum_{p\in \Omega_{\mathrm{ij}}}\left(1-\;\theta_{\mathrm{ij}}^{p}\;\varepsilon_{j}\right),$$ (6b)

$$\varepsilon_{j}=\;\frac{\log\;N_{j}}{\log\;N_{\mathrm{DS}}},$$ (6c)

and

$$\theta_{\mathrm{ij}}^{p}=\;\frac{\log\;{\bar{N}}_{j}\left(r_{\mathrm{ij}}^{p}\right)}{\log\;N_{j}}.$$ (6d)

The rate $\nu_{\mathrm{ij}}$ of values $S_{\mathrm{ij}}$ defined by Eqs 5a, 6b is equal to the rate of the corresponding correction factors $\vartheta_{\mathrm{ij}}^{\prime }$ and $\vartheta_{j}$:

$$\nu_{\mathrm{ij}}=\frac{\vartheta_{\mathrm{ij}}^{\prime }}{\vartheta_{j}}=\;\frac{1}{N_{\mathrm{ij}}}\underset{p\in \Omega_{\mathrm{ij}}}{\sum }\frac{1-\;\theta_{\mathrm{ij}}^{p}\;\varepsilon_{j}}{1-\;\varepsilon_{j}}\;=1+\frac{\varepsilon_{j}}{\left(1-\;\varepsilon_{j}\right)}\left(1-\frac{1}{N_{\mathrm{ij}}}\underset{p\in \Omega_{\mathrm{ij}}}{\sum }\theta_{\mathrm{ij}}^{p}\right).$$ (7)

Since $\;0\leq {\bar{N}}_{j}\left(r_{\mathrm{ij}}^{p}\right)\leq N_{j}$ for all $p\in \Omega_{\mathrm{ij}}$ , $0<\theta_{\mathrm{ij}}^{p}\leq 1$ for all $p\in \Omega_{\mathrm{ij}}$ **.** Therefore, $\nu_{ij}\;\geq 1$ **.**

**FIGURE 10:**
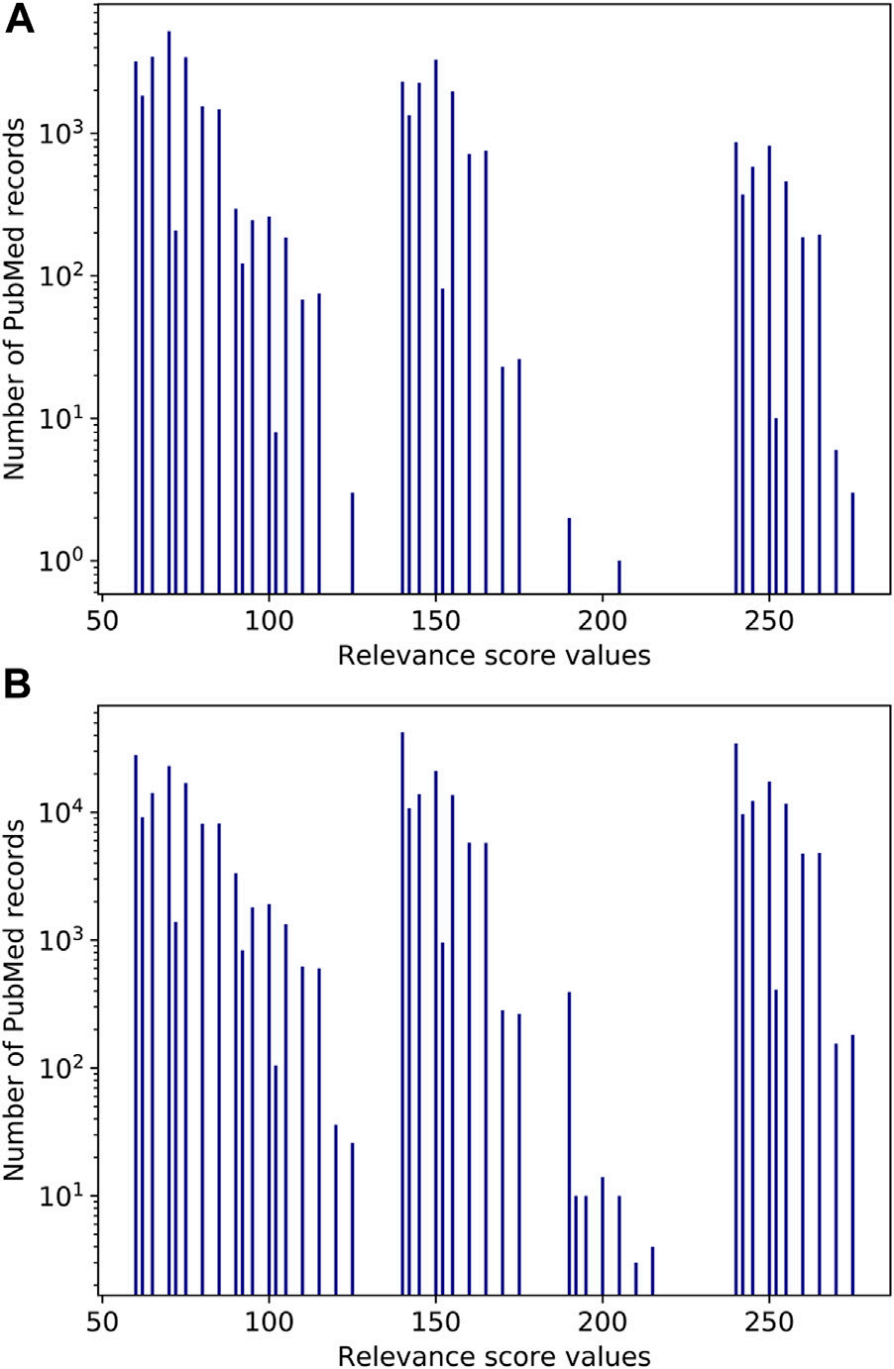
Histograms of the relevance score values for D-glucose (A) in PubMed records where D-glucose is co-mentioned with cholesterol, (B) in PubMed records where D-glucose is co-mentioned with any PubChem compound.

To illustrate calculation of the co-occurrence score using Eq.6a, consider D-glucose (CID: 5793) as a neighbor of cholesterol (CID: 5997). There are 37.8K PubMed records where cholesterol and D-glucose are co-mentioned among 447.6K PubMed records where D-glucose is mentioned. $\varepsilon_{j}$ value for D-glucose is 0.80, and the value of the correction factor is 0.20. The value of rate $\nu_{\mathrm{ij}}\;$depends on the counts of PubMed records for the values of the relevance score in two sets of PubMed records: the set of records where D-glucose was co-mentioned with cholesterol and the set of records where D-glucose was mentioned with any PubChem compounds (if D-glucose is co-mentioned with several compounds, the maximal value of the relevance score is taken). Corresponding bar plots are shown in Figure 10. The resulting $\nu_{\mathrm{ij}}$ value is 1.18, and the value of the correction factor $\vartheta_{\mathrm{ij}}^{'}$ is 0.24.

As explained in *Redundancy elimination*, a representative compound from each group of compounds with the same parent connectivity is selected to avoid clogging the knowledge panels with redundant information. Among the 294.6K CIDs matched in PubMed records, 101.1K CIDs (34%) have the same parent connectivity as another matched compound, forming 31.5K groups. The remaining 193.5K CIDs (66%) are singletons. This results in a total of 225.0K CID groups (i.e., 31.5K multi-CID groups plus 193.5K single-CID groups), from each of which a representative CID is selected to generate non-redundant chemical neighbors. The largest group contains 191 CIDs, which correspond to citric acid (CID 311) and its various salt forms (sodium citrate, calcium citrate, potassium citrate, and so on).

## Discussion

The PubChem literature knowledge panels, implemented in the PubChem Compound, Gene, and Protein pages, serve as an exploratory tool showing several most-related, non-redundant entities co-mentioned in the biomedical literature for the respective record being viewed (the query entity), along with a few most relevant PubMed records. The panels help the user to rapidly discover important relationships between chemicals, genes, proteins, and diseases, and quicky get a sense of the relationships in a set of papers. It is especially beneficial when a dataset is too big to examine. A sample of PubMed records co-mentioning the entities helps the user to understand the nature and reliability of the relationship. In addition, the user can download the list of the papers with a relationship of interest and read them to gain a deeper understanding.

The limitations of the approach used to develop the knowledge panels include the limitation of the current co-occurrence model itself as well as limitations of the technology employed for named-entity recognition and database matching. While an approach based on named entity co-occurrence in PubChem records is a useful data exploration tool, it is based on a simple well-known linguistic model. It is reasonable to think that even more sophisticated models may produce improved results.

A dictionary-based approach and the term matching procedures we use suffers from ambiguities when the same word could have multiple meanings and multiple matches. For example, lead has multiple meanings in common English besides being a synonym for the compound with CID: 5352425. Retinal is a synonym for the compound with CID: 638015 and also an anatomical term related to various retinal diseases under MeSH ID: D012164. CAT is a gene symbol for catalase (NCBI Gene ID: 847), a common name of organism domestic cat with NCBI taxonomy ID: 9685, and computer-assisted tomography under MeSH ID: D014057. MP2 is a synonym for the compound with CID: 15942661, a gene symbol for maturation polypeptide (NCBI Gene ID: 547827), an abbreviation for the second-order Møller–Plesset perturbation theory, as well a video file format, also known as MPEG-2. Approaches to mitigate ambiguities within the dictionary-based approaches include placing the term in a case-sensitive dictionary, deciding to always assign “the most common” meaning, or placing the term in a negative dictionary. However, in many situations the meaning is context-dependent, and novel disambiguation methods able to resolve context-sensitive situations are required. We are examing algorithms and methods that would enable us to better understand and utilize the contextual meaning of ambiguous terms. As with many other knowledge resources, the PubChem approach could benefit from incorporating information from more trusted, human-curated data sources. Additional curated information will allow to further cross-validate the data and promote trustful and reliable information through improved scoring.

Currently, the co-occurrence scores used for the knowledge panels are evaluated using Eq. 3, but we are working with more advanced scoring schemes, such as that given in Eq. 4, as well as incorporating validation scoring. Handling of (near-)redundancy in the neighbor lists of an entity is also an important issue to address in future development. In particular, the chemical name–structure association is an important area for improvement. For various reasons, a chemical name is often associated with multiple chemical structures that slightly differ from each other (e.g., in terms of: stereochemistry, isotopic composition, resonance forms, tautomeric structures, mixture/salt forms, etc.) (Hahnke et al., 2018). While PubChem chemical structure and chemical name processing attempts to handle such issues, it is imperfect. As a result, a single pair of chemical names, each of which can be mapped to multiple CIDs, often lead to many structurally similar CID-CID pairs, increasing the redundancy in neighbor relationships between chemicals. An improved algorithm to select good representative structures for chemical names would enhance the handling of this type of redundancy.

It may also be interesting to consider whether to automatically annotate a broader disease term (e.g., cancer) when its more specific disease name (e.g., breast cancer) is annotated in a PubMed article. While such extended annotations to broader disease terms would help to discover new relationships between entities, it would also increase the neighbor relationship redundancy.

PubMed records contain the title, abstract, and a few other metadata for publications in biomedical and life sciences. To support broader scientific communities, we are working toward extending our approaches beyond PubMed records. This may include papers published in scientific domains that are not well-covered by PubMed (such as chemistry, physics, material science, and nanotechnology) or full-text articles available in PubMed Central and other public repositories (such as government reports or curated text annotation). Patent documents are also of great interest. However, the extension of the approach to highly heterogeneous data sets requires a deep understanding of the relative importance of data and re-engineering of the scoring schemes and data representation.

In all, we believe the effort described here provides an effective and efficient means for users to quickly and efficiently understand the key biomedical entities associated to a given PubChem record. The user can rapidly explore a set of relevant PubMed papers for the set of associated entities (chemicals, genes/proteins, or diseases). The downloadable content empowers users to explore and analyze further the provided links. While many improvements can be made, it is already very helpful to and popular with users.

## Data Availability

The original contributions presented in the study are included in the article/supplementary material, further inquiries can be directed to the corresponding author.

## Abbreviations

CID, PubChem Compound Identifier (an integer number); PMID, PubMed Identifier (an integer number); MeSH, Medical Subject Headings; OPSIN, Open Parser for Systematic IUPAC Nomenclature; IUPAC, International Union of Pure and Applied Chemistry; HUGO, Human Genome Organization; HGNC, HUGO Gene Nomenclature Committee; IUPHAR, International Union of Basic and Clinical Pharmacology; BPS, British Pharmacological Society; EC number, Enzyme Commission number; TF-IDF, term frequency–inverse document frequency; NLP, Natural Language Processing; JSON, JavaScript Object Notation; XML, eXtensible Markup Language; CSV, Comma-separated values; ASNT, Abstract Syntax Notation Text.
